# Developing service models for adult palliative and end of life care for people with a learning disability (The DAPPLE Project): protocol for a mixed-method study

**DOI:** 10.1136/bmjopen-2025-106752

**Published:** 2025-07-25

**Authors:** Irene Tuffrey-Wijne, Elizabeth Tilley, Freya Tyrer, Zoebia Islam, Erica Borgstrom, Joanne Jordan, Gyles Glover, Louise Wallace, Christina Roberts, Jo Giles, Richard Keagan-Bull, Amanda Cresswell, Rebecca Anderson-Kittow

**Affiliations:** 1 Faculty of Health, Science, Social Care and Education, Kingston University, Kingston upon Thames, UK; 2 School of Health, Wellbeing and Social Care, The Open University, Milton Keynes, UK; 3 Leicester Real World Evidence Unit, University of Leicester, Leicester, UK; 4 LOROS, Hospice Care for Leicester, Leicester, UK; 5 University of Leicester, Leicester, UK

**Keywords:** PALLIATIVE CARE, Health Equity, Health Services Accessibility, Person-Centered Care, Quality Improvement

## Abstract

**Abstract:**

**Introduction:**

People with a learning disability face significant health and mortality inequalities as well as wider systemic inequities. Challenges in palliative and end of life care (PEOLC) include communication difficulties, lack of involvement in decision-making and multimorbidity. Early identification of PEOLC needs is challenging, impacting timely care planning. The study aims to (1) understand barriers and enablers to providing high-quality, accessible PEOLC for people with a learning disability, and identify effective service delivery models and interventions and (2) improve PEOLC quality and accessibility by developing robust guidance for health and social care services.

**Methods and analysis:**

This is a mixed-methods study guided by the NHS England 2021 Ambitions Framework and adopting the Social Model of Disability. There are four workstreams: (1) a retrospective cohort analysis of the Clinical Practice Research Datalink; (2) a rapid scoping review; (3) field work in four study sites across England, involving (a) interviews with senior leaders and commissioners (n=up to 16) and informal stakeholder engagement conversations; (b) ethnographic case studies with people with a learning disability at the end of life (n=up to 20) and retrospective case reviews of people with a learning disability who have died (n=up to 40), using family and staff interviews and (c) development and piloting of methods for enabling systematic identification of PEOLC need, using experience-based co-design and (4) patient and public involvement (PPI) activities and a co-production group of 10 people with a learning disability to support data analysis and outputs. Data will be analysed using adapted framework analysis methodology. This is an inclusive, co-produced study with significant involvement of advisors and researchers with a learning disability as part of the study team.

**Ethics and dissemination:**

Ethical approval has been obtained for workstreams 1, 3a and 3b. Significant attention has been paid to ensuring informed consent, making adjustments for capacity. Accessible information and consent forms will be used, involving consultees and adhering to the Mental Capacity Act for participants who lack capacity. Data security will follow General Data Protection Regulation rules. Dissemination will include patient exemplars, guidance and various resources, engaging stakeholders through multiple formats.

**Study registration:**

researchregistry10500.

**Strengths and limitations of this study**

- Meaningful inclusion and involvement of people with a learning disability as salaried researchers and members of a co-production group throughout the study.
- Multiple work packages allow for cross-referencing and synthesising evidence from various sources.
- Ethnographic case study methodology allows for the inclusion of people with severe and profound learning disabilities as study participants, who have been missing from the evidence base.
- Inclusion of study sites in diverse areas with regard to urban/rural setting, ethnic diversity and service engagement with delivering palliative and end of life care to people with a learning disability.
- A full comparative economic analysis, with cost-benefit assessment of different models of care, is beyond the scope of this study.

## Introduction

### Background

 There are around one million people with a learning disability in England (2% of the population),^1^ many of whom have complex health and social care needs. This study uses the term ‘learning disability’ as it is the current standard terminology within UK health and social care services; it is synonymous with the term ‘intellectual disability’, which is more widely used internationally.

People with a learning disability die on average 22 years earlier than the general population and are much more likely to die in hospital (62% vs 42%).^2^ In 2023, 42% of deaths of people with a learning disability in England were rated as ‘avoidable’, compared with 22% for the general population.^3^ Inequalities and avoidable mortality stem from factors associated with the social determinants of health for people with a learning disability (eg, dependence on others for noticing and dealing with health problems; poor health literacy); multiple comorbidities (people with a learning disability have an average of eight long-term health conditions at their time of death)^4^ and failings in healthcare delivery.^5^ The stark health and mortality inequalities^5 6^ were highlighted during the COVID-19 pandemic, with people with a learning disability dying at six times the rate of the general population.^7^

When people with a learning disability reach the end of life, they need to be well supported in a way that meets their needs and is in line with their wishes. Growing numbers of people with a learning disability now live into adulthood, including those with profound and multiple disabilities, with highly individual palliative care needs that may not have previously been the concern of adult services. Increased life expectancy for this population has also been associated with the onset of complex long-term conditions such as dementia, diabetes and frailty that require specialist support. Challenges in providing palliative and end of life care (PEOLC) for people with a learning disability include: difficulties with communication which affect all aspects of palliative care provision, including pain and symptom assessment; difficulties with patient insight into the condition, its treatment and possible outcomes; lack of involvement in end-of-life decision-making; multimorbidity and polypharmacy; complex social circumstances involving families as well as care staff; lack of reasonable adjustments to care; transitions in care settings; lack of experience among healthcare staff of people with a learning disability and lack of experience among learning disability staff of illness, death and dying, leading to fear and avoidance.^68^,^12^ Staff providing PEOLC to people with a learning disability have significant training needs.^13^,^15^ On a wider service level, challenges have also been noted regarding service collaboration, staffing levels, funding and management support.^16 17^ A range of national policy makers and advisors have recognised unacceptable inequities in PEOLC provision for people with a learning disability (eg,National Institute for Health and Care Excellence (NICE),^18^ Care Quality Commission,^19^ the Ambitions Framework,^20^ Hospice UK^21^).

Lack of timely identification of people who may benefit is seen as one of the greatest barriers to early palliative care.^18^,^22^ However, identifying when someone with a learning disability approaches the last year of life is particularly difficult, especially given the challenges in communicating and noting signs of ill-health and frailty, multiple comorbidities that may go unnoticed,^8 23^ or diagnostic overshadowing, where signs of ill-health are attributed to the learning disability.^24^ An investigation of 222 deaths of people with a learning disability supported by UK learning disability service providers found that less than a third of these deaths were anticipated by staff.^25^ This has a significant impact on the ability to plan for PEOLC and connect with PEOLC services in a timely manner.^21 23 25^

This difficulty has been further highlighted in our recent study on involving people with a learning disability in PEOLC planning.^26 27^ Planning for end-of-life support was hindered by the lack of insight into appropriate triggers for end-of-life care planning among health and social care professionals and informal carers. NICE recommends using identification tools such as the Gold Standards Framework^28^ or the Supportive and Palliative Care Indicators Tool,^29^ but these have not been validated for people with a learning disability and may not be suitable for this population.^30^ There have been some early attempts at developing learning disability-specific tools, but this needs further investigation.^23 31^

Empirical evidence on PEOLC provision for people with a learning disability is extremely limited. Largely anecdotal evidence from practice initiatives in the UK to improve PEOLC provision to people with a learning disability shows that good practice is over-dependent on committed individuals and not embedded within policies and organisational cultures.^32^ There have been no studies into the availability, nature and effectiveness of service models or interventions. There has been a lack of involvement of people with a learning disability in PEOLC research. The current evidence base is insufficient for the development of interventions and does not yet meet the requirements of the developmental first stage of the Complex Interventions Framework.^33^

### Building on previous studies

This project builds on over two decades of the principal investigator’s inclusive research around PEOLC for people with a learning disability, demonstrating that doing research with people with a learning disability around death and dying is not only feasible, but welcomed. It has grown out of extensive discussions with those affected, including learning disability service providers, family carer groups, self-advocacy groups of people with a learning disability, national policy makers and others. In particular, the research questions have been articulated following two NIHR-funded studies: NIHR202963 (2022–2024) on end-of-life care planning with people with a learning disability^26 27^; and NIHR129491 (2020–2023) on the support needed for older people with a learning disability and family carers.^34^ Previous research in barriers to PEOLC, focusing on ethnicity, is also relevant (NIHR17/05/30).^35^

This paper follows the Standards for Reporting Qualitative Research.^36^

### Aims and objectives

The study aim is to improve the quality and accessibility of PEOLC for people with a learning disability by producing robust guidance for health and social care services, with recommendations and accessible resources.

The study objectives are:

1. Map PEOLC trajectories of people with a learning disability, using national electronic health record databases.
2. Develop an understanding of existing evidence.
3. Explore, compare and contrast PEOLC services for people with a learning disability, with regards to (a) current models of care, commissioning practices and service coordination; (b) timely identification of PEOLC need and (c) individualised PEOLC provision/interventions for people with a learning disability.
4. Co-produce actionable recommendations and resources for service providers and commissioners, including interventions for the timely identification of PEOLC need.
5. Build capacity and produce guidance for future inclusive research with people with a learning disability.

The research questions are:

- What are the service delivery models and interventions within health and social care services that (a) enable the timely identification of the PEOLC needs of people with a learning disability and (b) are effective in meeting those needs?
- Within a range of service exemplars, what are the barriers and enablers to providing accessible, high-quality PEOLC to people with a learning disability; and what are the replicable elements of good practice?

## Methods and analysis

### Study team

This is a co-produced research project, using mixed methods. The developing service models for adult palliative and end of life care for people with a learning disability (DAPPLE) project team consists of researchers, including three university-employed researchers with a learning disability, from three universities and a hospice. They have a strong and complementary mix of skills, knowledge and expertise, including learning disability and palliative care research, translating research into policy, practice guidance and training resources, involving people with a learning disability and families in research, and innovative dissemination strategies.

### Theoretical framework

The DAPPLE Project is underpinned by the National Health Service (NHS) England and partners’ 2021 Ambitions Framework,^20^ building on the 2019 NICE guideline,^18^ which set out a vision to improve end-of-life care throughout England. It sets out six areas of importance in achieving excellence: (a) individualised care, (b) fair access to care, (c) maximising comfort and well-being, (d) coordinated care, (e) preparedness of staff and (f) preparedness of communities.

Our study assumes that this framework provides the gold standard of PEOLC. The research questions and methodological approaches are guided by the Ambition Framework, which is underpinned by eight foundations. We investigate how and to what extent these eight foundations are present with regards to people with a learning disability, and/or how they might be better achieved: (1) personalised care planning, (2) shared records, (3) evidence and information, (4) involving, supporting and caring for those important to the dying person, (5) education and training, (6) 24/7 access, (7) co-design and (8) leadership.

Our research is underpinned by the Social Model of Disability,^37^ a conceptual approach that sets out how people are disabled primarily by social and environmental factors, as opposed to individual physical or intellectual differences. The Social Model seeks to change society and systems to accommodate disabled people in order to address structural inequalities, for example, unequal access to healthcare. Our theoretical construct is that care is a situated practice, located in a nexus of healthcare infrastructure, interpersonal relationships, personal lived experiences and wider societal contexts. As such, to understand it, we cannot rely on only verbal accounts or single perspectives, nor do we assume definitive triangulation is possible. Instead, the aim is to understand as best as possible how things occur and with what consequences, being open to unanticipated and unexpected interpretations.

Our approach is focused on collecting data to describe the ‘what’ in a connected way that highlights issues and multiple understandings of power, structure and care—rather than a purely realist approach that has a stronger emphasis on identifying causal mechanisms for ‘what works’.

### Methods

The first three objectives are addressed in three distinct work streams, each with a different methodology: (1) national database analysis; (2) a rapid scoping review and (3) case studies in four study sites. Objectives 4 and 5 are met through the inclusive research processes, in particular the involvement of the co-production group of people with learning disabilities.

#### National data base analysis: a retrospective cohort study using electronic health records (objective 1)

##### Aims

To compare people with and without a learning disability to detect inequalities with regards to registration of PEOLC need and subsequent survival time; primary care and hospital care utilisation; palliative care referrals.

##### Methods

A retrospective electronic health record cohort study of people on the Clinical Practice Research Datalink (CPRD) in England aged 18+ years between 1 January 2010 to the last available update (includes 87 790 people with a learning disability). Data will be linked to hospital episode statistics, Office for National Statistics mortality data and deprivation data (approximately 75% of General Practice (GP) surgeries in England agree to the linkage scheme).

##### Sample size

A feasibility count conducted in April 2024 on people with and without learning disabilities and end-of-life care records given a sample population size of 1.7 million adults who meet the eligibility criteria, including 15 247 adults who have a learning disability. In the learning disability population, 3126 (21%) individuals have an end-of-life care record. This is a sufficient sample size.

##### Analysis

We will describe the characteristics of the population for all covariates under investigation by learning disability status, using means (SD; continuous covariates) and numbers (%; binary/categorical covariates).

### Rapid scoping review (objective 2)

#### Aims

To develop an understanding of existing evidence about service models and interventions for PEOLC for adults with a learning disability, with regards to (a) identifying need, (b) assessing and meeting need and (c) coordination and delivery of local services including professional development.

#### Methods

We will use a systematic approach to scope extant evidence drawn from diverse sources and using varied means of development, incorporating empirical research and grey literature.^16^ We will include international material, published in English from 2007. Given that our review constitutes an early component of a larger programme of work, it requires timely completion. A rapid scoping review is thus recommended. Conduct of the review will follow expert guidance for rapid scoping reviews^38^ and it will be reported according to the Preferred Reporting Items for Systematic Reviews and Meta-Analysis (PRISMA) extension for Scoping Reviews.^39^

We will search for grey literature using database and supplementary methods; the latter will also be used to augment searching for published literature. After de-duplication, records returned from database searching will be imported into the Rayyan database.^40^ Two reviewers will independently screen using titles and abstracts (where available) against explicit inclusion criteria. Full-text copies of evidence not excluded at this stage will be read by two reviewers. At both initial screening and full-text review, any discrepancies will be discussed by the two reviewers and, if necessary, with the wider review team. All sources of evidence excluded on the basis of full-text review will be recorded, alongside the reasons for exclusion. Using a PRISMA flowchart,^41^ we will record the process of screening to full-text inclusion.

#### Analysis and synthesis

The likely diversity of included evidence necessitates a flexible, but robust, approach to synthesis. We will summarise key characteristics of included evidence in a table of characteristics and identify patterns and trends in its volume, focus and content. We will synthesise findings using a narrative approach,^42^ appropriate when findings are derived from varying sources (research and non-research). We will aim to interpret these findings to generate new conceptual understanding, captured in analytical themes and constituent subthemes. Synthesis will be led by two researchers; all drafts will be shared with the research team and selected drafts with the Project Advisory Group to promote full analytical insight, strategic relevance and clarity.

### Case studies in four study sites (objective 3)

This is the central work stream, with data collection in four geographical areas in England (‘Study Sites’): Croydon (South London), Milton Keynes, Leicester and Kirklees (West Yorkshire). We have identified one or two study site partner organisations at each site: a hospice, a learning disability service provider, or both.

#### Aims

To identify barriers, enablers and good practice exemplars for the systematic delivery of timely, appropriate, individualised and coordinated PEOLC. The objectives are to explore, compare and contrast PEOLC services for people with a learning disability at four study sites, including models of care, identification of need and individualised PEOLC provision; and develop methods for timely identification of PEOLC need. There are three main sets of interlinked research questions, each with a distinct methodology, related to (a) models of care; (b) delivery of PEOLC and (c) developing methods for identification of need.

##### Models of care (objective 3a)

Research questions: What models of care can be implemented by health and social care services to ensure that the eight Foundations are in place locally? What are the local system approaches and strategic/commissioning priorities and practice in relation to PEOLC for people with a learning disability? What are the barriers and enablers of professional development and cross-organisational collaboration that promotes effective and timely delivery of PEOLC to people with a learning disability?

Methodology: Semistructured interviews with commissioners and senior managers in learning disability and palliative care services (n=8; each participant will be interviewed twice, at the start and end of the study), and scoping visits with informal stakeholder conversations (n=up to 12) and local stakeholder engagement events (n=up to 12).

Sample and recruitment: Inclusion criteria are: senior service managers of a service provider organisation, senior NHS commissioners or social care managers with responsibility for adults with a learning disability and/or palliative care services. Potential participants will be identified with support from our study site partners and members of the study team with pre-existing contacts among service providers. The purpose is to obtain an overview of provision and key issues in the local area; two participants within each local area will be able to provide this.

Inclusion criteria are: senior service managers of a service provider organisation, senior NHS commissioners or social care managers with responsibility for adults with a learning disability and/or palliative care services. Potential participants will be identified with support from our study site partners and members of the study team with pre-existing contacts among service providers. The purpose is to obtain an overview of provision and key issues in the local area; two participants within each local area will be able to provide this.

##### Delivery of PEOLC to people with a learning disability (objective 3b)

Research questions: What do people with a learning disability, families and carers perceive as their care and support need? What is the nature of PEOLC service delivery to adults with a learning disability? How is this experienced by people with a learning disability, families, carers and professionals? What do they see as barriers and enablers to good PEOLC?

Methodology: Case studies, using ethnography. Within each study site, we will undertake the following areas of work:

- Case studies of people with a learning disability currently receiving PEOLC or expected to be at end of life (n=up to 20), using ethnographic methods. This will include: (a) participant observation, recorded as detailed field notes (up to 420 hours). This involves a researcher spending time with the person with a learning disability. Exact timings and duration of data collection sessions will be agreed locally and with the participants and will depend partly on the participants’ health situation. We aim to spend time with participants in a variety of contexts, including at home, medical appointments and in-patient settings; (b) informal conversations with people with learning disabilities, family, carers, peers, managers and professionals, recorded as detailed field notes (n=up to 200). This is to explore perception of need, experience of service provision, challenges, what works well and what is difficult and (c) case file analysis where possible (n=up to 20).
- Retrospective case studies of people with a learning disability who had non-sudden deaths in the past 12 months (n=up to 40). We will conduct semistructured interviews with family/carers/managers/professionals (n=up to 120), recorded and transcribed, exploring similar questions as in the ethnographic case studies; and case file analysis where possible (n=up to 20).

Sample and recruitment: We will work closely with the study site collaborators, including local hospices and learning disability service providers. They will provide important local knowledge and participant access as required within sampling strategies. A 9-month period leading up to recruitment will allow for the research team to build contacts, relationships and trust with local services and staff. Potential participants will be identified by one of these contacts within the local service providers and approached by them (a ‘gatekeeper’) who will pass on the study information with an invitation to contact the research team if interested. Potential participants may also be identified through GP databases. All participants must live (or have died) within the geographical boundaries of the study sites.

Inclusion criteria for participants in the ethnographic case studies: Adults with a learning disability (mild, moderate or severe/profound), plus any one of the following: (1) recognised PEOLC need (for example, on the EOLC register, or receiving a PEOLC service), (2) advanced life limiting illness (eg,cancer, dementia) and (3) negative answer from formal carers to Surprise Question (“Would you be surprised if they died within 12 months?”).^43^

Inclusion criteria for participants in the retrospective case studies: Family, carer, staff or peer who was closely involved in the care and support of someone with a learning disability who died in the previous 12 months, and whose death was non-sudden. Excluded are deaths that were sudden and unexpected without a recognised PEOLC need.

##### Developing methods for identification of need (objective 3c)

Research questions: How do GPs, learning disability services and specialist palliative care services currently identify PEOLC need? What are the processes, decision points, barriers and enablers of timely service delivery of PEOLC to local adults with a learning disability who have PEOLC need? What are workable methods for identifying PEOLC need in local populations?

Methodology: Experience-Based Co-Design.^44^ We will recruit a co-production group from across the four study sites (n=8–10 people with a learning disability, family carers and staff). The group will take part in eight online workshop sessions to consider the study’s preliminary findings with regards to identification of PEOLC need in people with a learning disability. Together, they will co-design practical methods tailored for use in local settings. These methods will then be piloted within the four study sites, with specific approaches shaped by the outcome of the co-design process.

### Data analysis

We will use inclusive data analysis and output development approaches with a focus on framework analysis and mind-mapping techniques.^45 46^ We have found that these work well with, and can be understood by, a diverse group of researchers, including co-researchers with a learning disability.^47 48^ After each data collection session (including all data types), key issues will be noted in a structured summary template with no formal coding. Sections include participant and data collection details, plus deductive headings (developed from the research questions) and inductive headings (to allow new issues to emerge). Further template sections will include key documents, observations, quotations (verbatim quotations will be added when transcripts are ready) and reflections. To bring teams together and ensure integration of data and learning across sites, there will be monthly online data analysis sessions with researchers across the four sites, to share development of themes, reflections and puzzles. We will also link the data analysis with the co-production group of people with a learning disability.

### Co-production, patient and public involvement and engagement (PPIE) and outputs

#### Aims

To ensure that PPIE, active involvement of people with a learning disability and a focus on equity, diversity and inclusion is embedded across the study; and to co-produce actionable recommendations, tools and resources for commissioners and health and social care providers.

#### Methods

A national group of up to 10 people with a learning disability will meet with the research team monthly online. They will consider emerging study findings and contribute to co-producing study outputs. On-going stakeholder engagement activities and co-production of outputs (see ‘Dissemination’), involving the entire research team, also sit within this workstream.

### Time frame

This 3-year study will be conducted between 1 September 2024 and 31 August 2027.

### Patient and public involvement statement

People with a learning disability were actively involved in the development of the protocol. The research team includes three university-employed researchers with a learning disability, contributing to the diversity and inclusivity of the team. Two of the researchers with a learning disability are grant co-applicants for the study and co-developed an accessible version of the protocol. This diverse and collaborative team will share power and responsibility throughout the project, including the generation of knowledge. We will also establish and work with a co-production group of 10 people with a learning disability, who will meet monthly online, and will actively advise on all aspects of the study, providing feedback on emerging findings and supporting the dissemination of outputs. Additionally, there is a Project Advisory Group providing strategic oversight and guidance. This group will include a wide range of stakeholders, such as people with a learning disability and family carers, as well as those with expertise in practice, academia and health and social care policy.

## Ethics and dissemination

### Ethics

Approval for the CPRD component for work stream 1 (national database analysis) has been obtained from CPRD’s Research Data Governance Process (protocol ID: 24_004154). Ethical approval for 3a (interviews with commissioners and senior service managers) has been obtained from Open University Human Ethics Committee, reference number 2024-0560-2. Approval for case studies (3b) has been obtained from the Research Health Authority (HRA) and Health and Care Research Wales, reference number 25/WM/0065. Ethical approval for the final stage of 3c (testing methods for identification of PEOLC need) will be sought from the HRA in 2026.

There are important ethical considerations with regards to informed consent, power imbalance, confidentiality and the management of distress around a sensitive research topic. Interview topic guides and observation guides will be included with associated information sheets (including in easy-read and video format), consent forms and de-briefs (including complaint procedures, or where to get help if questions or concerns arise). In the ethnographic field work (case studies), care will be taken to ensure that people are happy to be observed and have the researcher (and in some cases, co-researcher) spend time with them using ‘process consent’. This involves ensuring before and during each visit that the participant remains happy with the researcher’s presence. For those with communication difficulties, familiar accessible communication methods will be used. Researchers will pay close attention to issues of mental capacity, in accordance with the Mental Capacity Act 2005. This includes the decision whether to take part in research. If the person lacks capacity to make this decision at this time (which may be due to their learning disability or to the fact that they are ill), the advice of a consultee (someone who knows them well) will be sought. However, we will make every effort to maximise their capacity by using accessible information materials and consent forms, explained face to face with each participant by the researcher and, where appropriate, with someone who knows the participant well. General Data Protection Regulation legislation will be followed for data security and best practice in managing and anonymising data storage, analysis and outputs. Transcribed data will be anonymised and all identifiable features removed. All data will be stored on university secure systems, password protected and accessible only to the research team.

### Dissemination

We will produce rigorous and relevant evidence about how health and social care services can work together to assess and meet the needs of people with a learning disability at the end of life. Specific co-produced outputs include:

- A set of patient exemplars, highlighting positive practice, pathways and experiences. These will represent a range of different settings, contexts, profiles of people with a learning disability and illness/dying trajectories.
- Final methods and guidance for identification of PEOLC need will be made freely available for use within health and social care services, including learning disability services, specialist palliative care and primary care services.
- A series of online-hosted resources and outputs, including OpenLearn educational/training resources (an award-winning free educational platform with 16 million+annual visits).

We will make outputs available in a range of formats, including publications in peer-reviewed journals, webinars and conference presentations, plain English, easy read and video formats. We anticipate strong engagement with stakeholders throughout and beyond the study, through our website, webinars and social media activities.

To promote stakeholder engagement from the start of the study, there is a dedicated website (www.dappleproject.com) and social media accounts including Bluesky and LinkedIn. There will be regular blogs, mailing lists, specialist networks and webinars, and the co-production of outputs including academic papers. This protocol has been summarised not only in plain English, but also in easy-read and video format, which can be found on the project website.
